# Supplementary material for: Empirical methods for controlling false positives and estimating confidence in ChIP-Seq peaks
Source: BMC Bioinformatics. 2008 Dec 5;9:523. doi: 10.1186/1471-2105-9-523 (PMC2628906; doi:10.1186/1471-2105-9-523)
Supplement: Additional File 1 — A variety of html documents from the USeq web site detailing the available applications, their best usage, output file type descriptions, command line menus, etc. [file 1471-2105-9-523-S1.zip › USeqUserGuides/JavaDoc.zip!/JavaDoc/edu/utah/seq/compression/CompressFasta.html]

CompressFasta


|  |  |  |
| --- | --- | --- |
| **Overview** \| **Package** \| **Class** \| **Use** \| **Tree** \| **Deprecated** \| **Index** \| **Help** | |  |
| PREV CLASS   NEXT CLASS | **FRAMES**    **NO FRAMES**     **All Classes** |  |
| SUMMARY: NESTED \| FIELD \| CONSTR \| METHOD | DETAIL: FIELD \| CONSTR \| METHOD |  |


---


## edu.utah.seq.compression Class CompressFasta

```
java.lang.Object
  edu.utah.seq.compression.CompressFasta
```

---

``` public class CompressFasta extends java.lang.Object ```

Application that compresses or decompresses a multi fasta file.
Names of the individual reads will be converted to 1,2,3...
For compression, each of the reads must be 1) the same length 2) on one line 3) only contain gatc characters.
Thus this app is best suited for compressing signature sequencer reads.
It has a 20% better compression rate that just zipping, gzipping, or bzip2ing the text fasta.

---

**Constructor Summary**

`CompressFasta(java.io.File[] files)`


| **Method Summary** | |
| --- | --- |
| `boolean` | `compressIt(java.io.File fastaFile, java.io.File compressedFile, java.lang.String notes)` |
| `void` | `decompressIt(java.io.File f)` |
| `boolean` | `findSeqSizeAndNumberReads(java.io.File fastaFile)` |
| `static void` | `main(java.lang.String[] args)` |
| `boolean` | `printBinarySequence(java.io.File compressed, java.io.File decompressed)` |

**Methods inherited from class java.lang.Object**

`equals, getClass, hashCode, notify, notifyAll, toString, wait, wait, wait`

**Constructor Detail**

### CompressFasta

```
public CompressFasta(java.io.File[] files)
```


**Method Detail**

### main

```
public static void main(java.lang.String[] args)
```

---


### decompressIt

```
public void decompressIt(java.io.File f)
```

---


### findSeqSizeAndNumberReads

```
public boolean findSeqSizeAndNumberReads(java.io.File fastaFile)
```

---


### compressIt

```
public boolean compressIt(java.io.File fastaFile,
                          java.io.File compressedFile,
                          java.lang.String notes)
```

---


### printBinarySequence

```
public boolean printBinarySequence(java.io.File compressed,
                                   java.io.File decompressed)
```


---


|  |  |  |
| --- | --- | --- |
| **Overview** \| **Package** \| **Class** \| **Use** \| **Tree** \| **Deprecated** \| **Index** \| **Help** | |  |
| PREV CLASS   NEXT CLASS | **FRAMES**    **NO FRAMES**     **All Classes** |  |
| SUMMARY: NESTED \| FIELD \| CONSTR \| METHOD | DETAIL: FIELD \| CONSTR \| METHOD |  |


---
